# Restoration of posterior teeth by narrow diameter implants in hyperglycemic and normoglycemic patients – 4-year results of a case-control study

**DOI:** 10.1007/s00784-024-05786-0

**Published:** 2024-06-22

**Authors:** Daniel Diehl, Angelina Bespalov, Mehmet Selim Yildiz, Anton Friedmann

**Affiliations:** 1 Department of Periodontology, School of Dentistry, Faculty of Health, Witten/, Herdecke University, Alfred-Herrhausen Str. 45, 58455 Witten, Germany; 2 Institute of Pharmacology and Toxicology, Center for Biomedical Education and Research (ZBAF), Faculty of Health, Witten/Herdecke University, Stockumer Straße 10, 58453 Witten, Germany; 3 Department of Periodontology, Faculty of Dentistry, Altınbaş University, Zuhuratbaba, İncirli Cd. No:11-A, 34147 Bakırköy, Istanbul, Turkey

**Keywords:** Marginal bone loss, Type 2 diabetes mellitus, Narrow diameter implants, Bone loss

## Abstract

**Objectives:**

To investigate the four-year clinical outcome and marginal bone loss around narrow-diameter implants in patients with uncontrolled diabetes mellitus type 2 (T2DM) and normo-glycemic individuals.

**Materials and methods:**

In 11 T2DM patients with a concentration of glycated hemoglobin (HbA1C) > 6.5% (test group) and 15 normoglycemic patients (HbA1C < 6.0%; control group), one narrow-diameter tissue level implant, placed in the posterior maxilla or mandible, was investigated. The clinical parameters probing depth (PD), bleeding on probing (BOP), attachment loss (CAL), recession, and papilla bleeding index (PBI) were assessed manually after 24 and 48 months of function. The paired digital periapical radiographs were analyzed regarding the change in marginal bone level (MBL) from baseline to 48 months post-op. The technical complications were recorded.

**Results:**

In the T2DM group, 11 patients were available for follow-ups. The overall implant survival rate after 48 months was 100%. The differences in means for the clinical parameters and the MBL between the T2DM and normo-glycemic patients for the observation period were statistically non-significant. No technical complications were recorded.

**Conclusions:**

The study demonstrated an encouraging clinical outcome with ND implants in patients with uncontrolled T2DM compared to non-diabetics after 48 months’ post loading.

**Clinical relevance:**

Patients with HbA1C > 6.5% may benefit from the treatment with narrow-diameter implants by avoiding complex surgical interventions with augmentation procedures.

**Registration number (clinicaltrials.gov):**

NCT04630691

**Supplementary Information:**

The online version contains supplementary material available at 10.1007/s00784-024-05786-0.

## Introduction

Type 2 diabetes mellitus (T2DM) is a group of metabolic disorders characterized by high serum glucose levels due to insufficient insulin supply, defective receptor function, or both [1]. The cardinal symptom of type 2 diabetes is chronic hyperglycemia (CH), which leads to numerous secondary diseases. At the molecular level, so-called Advanced Glycation Endproducts (AGE), which consist of non-enzymatic glycation of proteins, are responsible for most diabetes-associated pathologies [2]. AGEs can cause cell damage by attaching to various receptors in the body, including the Receptor of Advanced Glycation Endproducts (RAGE). During chronic hyperglycemia, RAGE is expressed in many cell types, activating the transcription factor NF-kB. This transcription factor regulates the transcription of many genes related to the immune system and inflammatory responses, including those for inflammatory cytokines. Increased cytokine activity can cause persistent inflammation, negatively impacting wound healing and creating a self-sustaining inflammatory response commonly seen in diabetic wounds [3]. In hyperglycemic periodontitis patients, this is clinically expressed in significantly increased attachment loss [4]. Consequently, dental implants have also exhibited an elevated risk of early implant loss, peri-implantitis onset, and marginal bone loss [5–7].

The lack of sufficient dimensions of the alveolar ridge due to atrophy or periodontitis often prevents the installation of dental implants. For this reason, surgical augmentation measures are frequently required before implant placement [8]. However, extensive reconstructive procedures on the edentulous alveolar ridge may not always be an effective treatment method. As many clinical studies about bone augmentation name T2DM as an explicit exclusion criterion, there is little evidence regarding the success rates of various augmentation surgery interventions [9]. Nonetheless, given the pathophysiological consequences of uncontrolled T2DM, it seems evident that T2DM-related vascular and immunological repercussions pose a significant risk for augmentation procedures in the edentulous jaw [10].

Consequently, implant-related dental research has focused on reducing implant dimensions to circumvent extensive bone augmentation. In this regard, it has become evident that short implants may even exhibit better long-term results than normal implants in augmented bone [11]. Over a 5-year observation period, they have also been shown to be successful in patients with a history of periodontitis [12].

In analogy to the vertically reduced implant diameter, narrow-diameter implants (NDI) were designed to allow implant-supported restorations without the need for augmentation in the case of horizontally atrophic alveolar ridges. According to Schiegnitz and Al-Nawas, implants with a diameter of less than 3.5 mm and more than 2 mm are classified as NDI [13]. In 2014, a systematic literature review revealed that numerous clinical studies have demonstrated the effectiveness of NDI [14]. More recently, it has been found that NDIs work well in hyperglycemic patients, leading to successful clinical outcomes and osseointegration. This suggests that NDI could be a viable treatment option for T2DM patients with narrow ridge dimensions [15, 16]. However, long-term observational studies are warranted to investigate the behavior under chronic hyperglycemia. The aim of this follow-up to a case-control study was to assess the marginal bone level changes and clinical parameters around NDI placed in hyperglycemic and normoglycemic patients after an observation period of four years.

## Materials and methods

This is a 4-year follow-up analysis nested within a case-control study investigating the clinical performance and marginal bone loss of NDI in uncontrolled T2DM patients. The original study protocol is reported elsewhere [15, 16]. In brief, thirty-two patients aged between 53 and 82, with a mean age of 67, were initially enrolled. Sixteen patients known to suffer from T2DM and diagnosed with an HbA1_C_ > 6.5% were considered as uncontrolled hyperglycemic and assigned to the test group, whereas 16 non-diabetic patients (HbA1C ≤ 6.0%) were allocated as controls. HbA1_C_ was assessed by the patient`s general practitioner (GP), who submitted the results to our clinic. For this follow-up, the most recently measured HbA1c values were enquired from the GP. None of the measured values was older than 4 months. The Witten/Herdecke University Ethics Committee approved the study protocol (108/2012), and all participants signed the informed consent. The study treatment modalities complied with the Declaration of Helsinki and fulfilled the Good Clinical Practice (GCP) criteria.

### Exclusion criteria

- Immobility.
- Periodontal surgery and antibiotic therapy within the last six months before baseline.
- Pregnancy and lactation period.
- Full Mouth Plaque Score (FMPS) > 25%.
- Untreated periodontitis.
- Smoking > 10 cigarettes/day.
- Insufficient crestal width for NDI installment (horizontal dimensions < 5 mm).
- Previously performed ridge augmentation procedure for a staged implant placement.
- Permanent medication affecting blood perfusion rate and bone metabolism.

### Surgical procedure

In brief, each patient received one to a maximum of two 3.3 mm tissue level (RN TL) titanium-zirconium alloy implants (Roxolid®) with the SLActive® (Institut Straumann AG, CH) surface configuration. All implants were placed by two experienced periodontists according to the manufacturer’s surgical protocol instructions for transmucosal healing. The surgical approach was standardized: a mid-crestal incision in the edentulous area was combined with intrasulcular incisions in neighboring teeth while vertical releasing incisions were omitted. A buccal and a lingual flap were minimally reflected to have a clear view of the crest. The implant installment was performed under local anesthesia (Ultracain DS forte®- Sanofi-Aventis, Frankfurt, Germany). All implants were restored by either a single crown or a fixed partial denture (FPD), and all restorations used either SynOcta® or Variobase® abutments (Straumann®, Institut Straumann AG, CH). The restorative protocol allowed for both types of implant crown fixation, either screw-retained, or cementum-retained. If two implants were placed, the most posterior one served as the study implant for this patient. After the surgery, all implants were radiographically documented using the parallel technique for periapical X-rays. For post-operative management, patients were advised to use chlorhexidine (Chlorhexamed GlaxoSmithKline Consumer Healthcare GmbH & Co. KG, Munich, Germany) mouth rinse (0.2%) twice a day instead of mechanical biofilm removal in the treated area. No systemic antibiotics were prescribed, but analgesic medication was recommended based on individual needs. A follow-up visit after 3 days was scheduled, and sutures were removed after 7–10 days. Chipping of the porcelain coating, screw-loosening, de-cementation, fracture of any component, or other maintenance requirements was considered technical complications and recorded during the observation period.

### Assessment of clinical parameters

The peri-implant PD, CAL, and recession were estimated using a PCP-11 probe (Hu-Friedy, Tuttlingen, Germany) at four sites per implant. The measurements were carried out 24 months (T1) and 48 months after implant surgery (T2) on the integrated implants. Furthermore, the bleeding on probing (BOP) and papilla bleeding index (PBI) on the buccal aspect were investigated. All clinical parameters were assessed by one independent investigator who was not involved with the initial implant installation (A.B.).

### Marginal bone level assessment

For further radiographic analyses of MBL, the post-surgery digital radiograph was used as the baseline. At both T1 and T2, a digital radiograph was performed using the parallel technique. Each pair of radiographs was calibrated by estimating the distortion coefficient and adjusting the images to the given implant diameter, as reported earlier [15]. In brief, the distance between single threads of each implant projected on the standard monitor served to calculate the distortion coefficient in the vertical dimension. One calibrated investigator (M.S.Y.) performed all the analyses regarding MBL utilizing ImageJ2 software [13]. To assess the vertical bone dimension, two landmarks were identified on each radiograph to establish a reference level. These landmarks indicate the highest point of the crestal bone in contact with the implant at the beginning of the rough surface on both the mesial and distal aspects of the baseline radiograph. A perpendicular line along the implant axis was drawn between these landmarks, and the distance was calculated in mm. The measurements were repeated at T1 and T2, and the MBL was calculated following the formula

$$\Delta \mathrm{MBL}=\mathrm{MBL}_{\text {Baseline }}-\mathrm{MBL}_{\mathrm{T} 1 / \mathrm{T} 2}$$

### Statistical analysis

Quantitative variables were analyzed by calculating their mean and standard deviation. The statistical analysis was performed using Prism 9 software from GraphPad (San Diego, California). Raw data was tested for Gaussian distribution using the Kolmogorov-Smirnov test. Since all variables showed normal distribution, parametric tests were used for further analysis. The differences between study groups were evaluated by Student’s t-test for clinical parameters. A delta value (ΔMBL = MBLBaseline - MBLT1/T2) was calculated Student’s t-test for group comparisons of marginal bone level. The significance level was set at *p* = 0.05.

## Results

A total of 26 patients with a mean age of 63 were available for 4-year follow-up visits, i.e. 15 patients from the control group and 11 patients from the T2DM group (Table 1). Due to patient drop-outs in the test group, the mean HbA1c value increased to 7.35 (+-0.68) compared to the results cohort at the one-year evaluation [15]. No patient from the T2DM group was disqualified from the group allocation due to improved HbA1c. All restored study implants were under functional load after 24 and 48 months, resulting in an overall survival rate of 100% for both groups, respectively. No technical complications were observed. However, one patient from the T2DM group lost one 3.3 × 8 mm after 24 months of functional load, which was not the study implant. Three single crowns in two patients were screw-retained; all other crown or bridge frameworks were cemented using glass-ionomer luting cement (Ketac Cem, ESPE, Germany).

**Table 1:** Patient demographics

	All groups	Test	Control
Patients (initial)	26 (32)	11 (16)	15 (16)
Dropouts	6	5	1
Implants failed	0	0	0
Mean Age (Range)		67 (49-80)	59 (39-70)
Sex			
Male (%)	14(48.3%)	8 (61.5%)	6 (37.5%)
Female (%)	15 (51.7%)	5 (38.5%)	10 (62.5%)
Smoking	-	-	-
Mean HbAIC (±SD)	-	7.35 (±0.71)	-
Jaw			
- Maxilla	12	5	7
Mandibula	17	8	9
Implant Total	29	13	16
Implant length			
- 8 mm	7	4	9
-10 mm	15	6	4
12 mm	7	3	1
Framework			
Screw-retained	3	2	1
Cement-retained	26	11	15

**Fig. 1:**
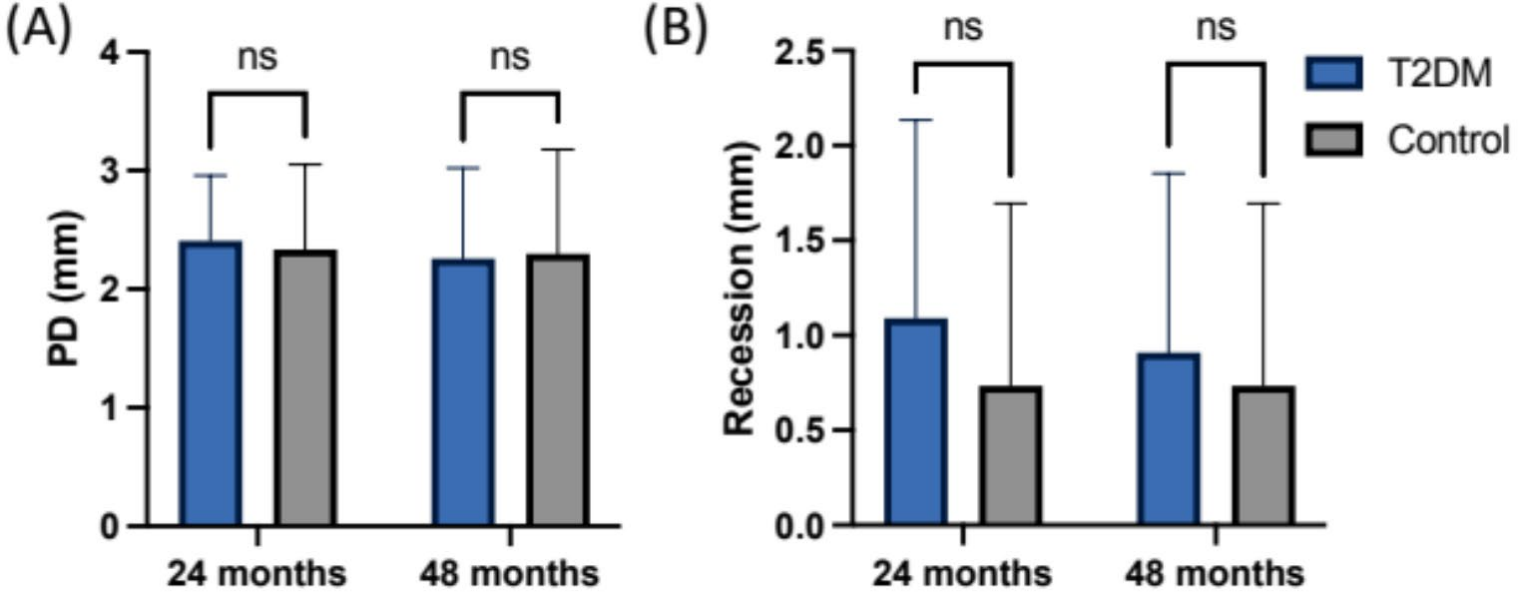
Results for statistical evaluation of clinical measurements at T1 and T2 (**A**) Clinical attachment loss (**B**) Recession. ns=not significant

**Fig. 2:**
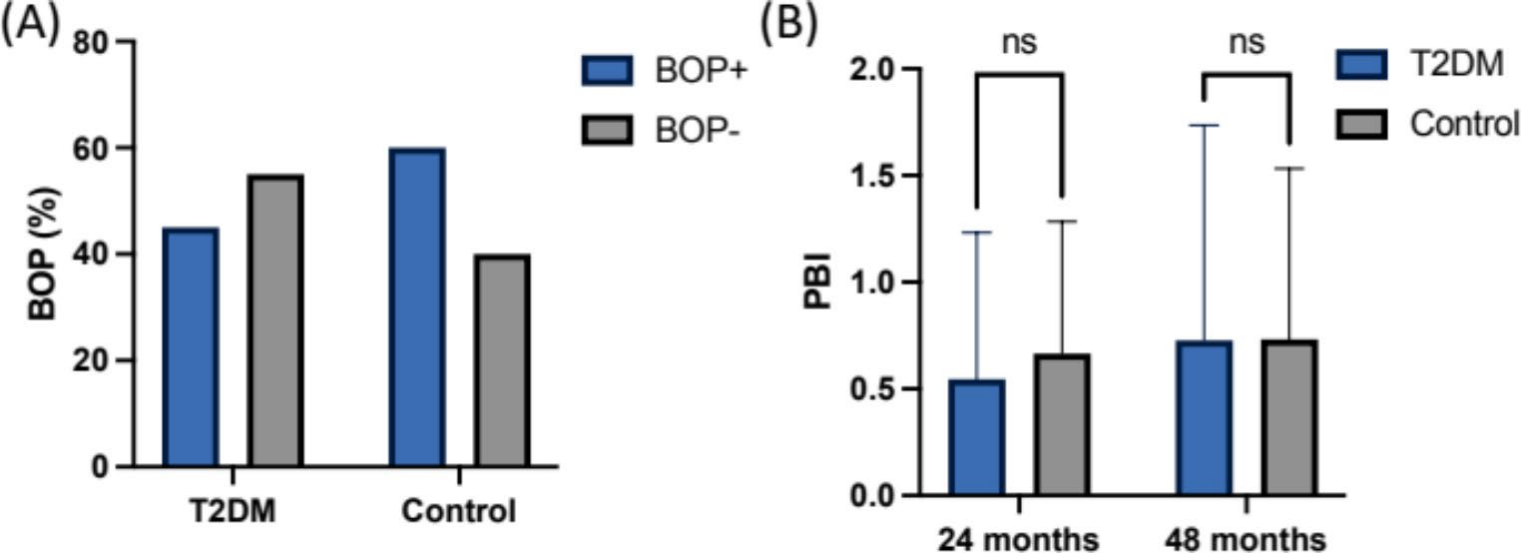
Descriptive statistics for **(A)** BOP and **(B)** papilla bleeding index (PBI)

The mean peri-implant PD was measured at 2.3 ± 0.7 mm (T1) and 2.3 ± 0.8 mm (T2) for the normoglycemic group, whereas the T2DM group exhibited a mean PD of 2.4 ± 0.6 (T1) and 2.3 ± 0.8 mm (T2) at the follow-up visits (Fig. 1). Thus, the mean values for the clinical parameters assessed after 24 and 48 months revealed statistically non-significant differences (*p* = 0.6 and *p* = 0.29, respectively, Table 2). The BOP index appeared slightly increased in the control compared to the T2DM group (60–45%) at T2, whereas the PBI remained indifferent (*p* = 0.99) in both groups (Fig. 2).

**Table 2:** Summary of all measurements taken during the study, including p-values

		T2DM	Control	*p*
Probing depth in mm (mean ± SD)	T1	2.4 ± 0.55	2.3 ± 0.71	0.959
	T2	2.3 ± 0.77	2.3 ± 0.88	0.987
Recession in mm (mean ± SD)	T1	1.1 ± 1.04	0.73 ± 0.96	0.591
	T2	0.91 ± 0.94	0.73 ± 0.96	0.880
ΔMBL (mm)	T2	0.09 ± 0.89	0.15 ± 0.83	0.875
BOP (% per group)		45%	60%	0.144
PBI (mean ± SD)		0.7 ± 1.01	0.7 ± 0.79	0.998

**Fig. 3:**
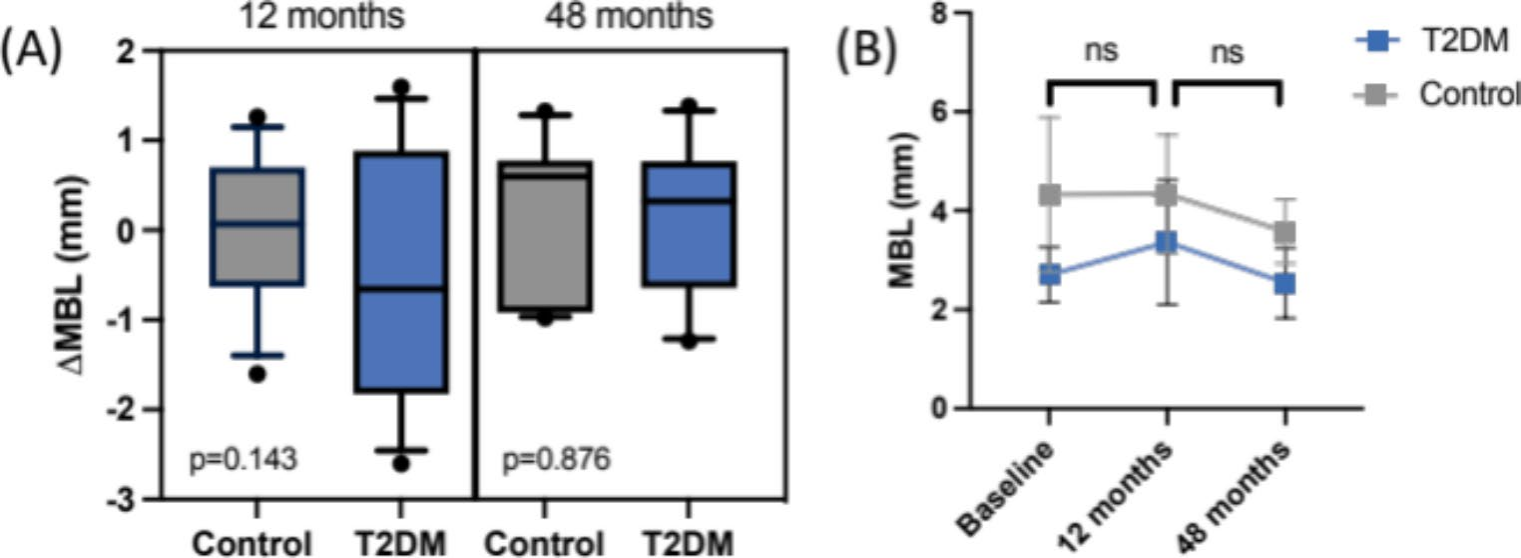
Results from radiographic evaluation of marginal bone loss (MBL). **(A)** Comparison of mean ΔMBL (**B)** Descriptive statistics of mean MBL measurements at baseline, after 1 year and after 4 years (T2)

The radiographic analysis revealed a non-significant marginal bone level (MBL) change for both study groups. However, one outlier was removed from the control group, as the MBL results revealed an MBL gain of more than 4 mm related to an insufficient angulation of the radiograph (Supplementary Fig. 1). The ΔMBL at T2 was calculated with 0.1 ± 0.89 mm for non-diabetic and 0.16 ± 0.83 mm for the T2DM patients. The comparison between both groups disclosed no significant differences (*p* = 0.876) (Fig. 3). Additionally, no significant differences in MBL were found between the values measured after 12 months [15] and T2, indicating no further bone level changes over the 4-year observation period.

## Discussion

Due to its diversity of possible systemic consequences, T2DM and chronic hyperglycemia remain a significant challenge in periodontal and implant research. Albeit the evidence has increased substantially for implant-related questions in T2DM patients, the outcomes for various types of augmentative surgery are yet to be investigated in an uncontrolled diabetic cohort. To date, available literature suggests keeping invasiveness in these patients at a minimum. Within this frame of reference, NDIs represent a promising treatment option in posterior areas presenting with diminished alveolar ridge dimension. However, adequately controlled long-term studies with this implant type in hyperglycemic patients are warranted.

According to the clinical literature, a successful implant is defined as an implant that doesn’t have any biological or technical complications or adverse aesthetic outcomes. To determine the biological success of an implant, bleeding on probing (BOP), peri-implant probing depth (PD), and radiographical marginal bone loss (MBL) is recommended. The MBL around implants in systemically healthy patients is estimated at 1.0–1.2 mm in the first year following insertion and constitutes one of the most important clinical and radiological parameters for the foreseeable long-term success of an implant [17–19]. Recently, we reported the clinical and radiographic outcomes of our study cohort one year after implant installment and found non-significant marginal bone loss of 0.66 mm, ranging among the expected values in this timeframe [15]. Al-Shibani et al. (2019) and Alsahhaf et al. (2019) conducted clinical studies to evaluate the clinical and radiological parameters of NDIs placed in diabetic and non-diabetic patients. Over a three-year period, Al-Shibani et al. found no significant differences in peri-implant indices or marginal bone loss. Conversely, Alsahhaf et al. observed increasing marginal bone loss in the first 3 years with rising HbA1c levels in diabetic patients, although this remained statistically non-significant [20, 21]. The results of our study seamlessly align with the published data from the above-mentioned authors. After 4 years, we also documented a measurable but statistically non-significant difference in MBL in the T2DM group, which was coupled with a 4-year success rate of 100%. However, due to different patient dropouts, we cannot make this claim for all implants originally enrolled in the study. Our findings were replicated in a prospective case-control study conducted by Cabrera-Dominguez et al. (2020) in patients with controlled type 2 diabetes mellitus. The study included 22 diabetics and 22 healthy controls who received the same type of implant as in our study. The study showed a high implant survival rate of 100%, with no significant differences in marginal bone loss, probing depth, or peri-implant bleeding [22]. However, the clinical literature exhibits high heterogeneity related to the different T2DM study cohorts. A recent retrospective analysis of implants placed up to 30 years ago revealed that diabetes does, in fact, have a negative influence on MBL. Interestingly, the MBL trajectory showed the most substantial differences after 10–30 years, indicating that notable differences in MBL may start appearing with our cohort in a couple of years’ time [23]. Moreover, a systematic review and meta-analysis clearly stated that T2DM patients exhibit significantly higher MBL than healthy patients, which is related to elevated levels of pro-inflammatory cytokines [24]. Given that inflammatory reactions of any kind are severely exacerbated in diabetic patients via the AGE/RAGE/NF-kB-axis, it appears reasonable to suggest that oral hygiene and the prevention of subclinical peri-implant inflammation, on the one hand, and individual glycemic control, on the other hand, may be the factors contributing to the large heterogeneity in the clinical literature. After all, the patients who were eligible for follow-ups remained strictly compliant with supportive periodontal therapy and control visits twice a year throughout the observation period.

Among inflammatory and systemic risk factors, the implant type and the location of the implant-abutment connection are important criteria with an evident influence on marginal bone loss [25]. The type of NDI used in this study exhibited a tissue-level (TL) neck configuration, where the connection is located supracrestally. Given that dental implants exhibit a fixed biologic width, TL implants have been suggested to exhibit more stable marginal bone levels [26, 27]. On the contrary, evidence for an inferior MBL behavior of TL implants compared to bone-level implants has emerged in recent years, suggesting that platform-switching may circumvent the previously made observations [28, 29]. However, marginal bone loss, as well as peri-implantitis onset, is also significantly influenced by the abutment design and restorative margin in bone-level implants [30]. Obviously, the clinical data remains heterogeneous, rendering any notion regarding the implant-abutment connection speculative at this point [31].

In this study, one implant in the control group and two implants in the test group were fitted with screw-retained crowns instead of cement-retained two-piece crowns, which may have introduced unwarranted variation to each of the groups. While earlier clinical studies noted more favorable soft tissue outcomes in screw-retained prostheses [32], no significant differences in terms of MBL between both types of restoration have ever been reported [33, 34]. Additionally, finite element analyses of both restoration methods showed that the restoration type does not influence the stress on peri-implant bone [35]. Moreover, a more recent retrospective study reported equal MBL for cemented restorations on tissue levels implants and screw-retained prostheses, indicating that the chosen restoration type may not necessarily influence the results of this study [36].

Nonetheless, this study has some limitations related to its design and sample size. Since it was conducted as a pilot study, an adequate sample size calculation was not possible at the beginning of this observational study. Because some participants dropped out of the test group, the statistical power of this report has been further diminished. Therefore, the results must be interpreted with sensible caution. Moreover, the observed but insignificant differences may potentially become significant in a larger study cohort. However, in the *post hoc* power analysis we performed after the one-year results, a sample size of at least 1000 patients would be required [15].

Taken together, the study reveals positive clinical outcomes for NDI in patients with poorly controlled T2DM and normoglycemic patients after a four-year period. The peri-implant tissues and implant-borne restorations show similar biological responses and functions for the NDIs in both, hyperglycemic and normoglycemic patients. These observations indicate that the installation of NDI resulted in predictable peri-implant bone loss and clinical parameters over a medium-term observation period, regardless of the glycemic condition.

## Conclusions

In terms of short-term implant success and implant survival, there were no statistically significant differences between normoglycemic and diabetic patients, underlining that tissue-level NDIs pose a minimal-invasive and predictable treatment option in T2DM patients.

### Electronic supplementary material

Below is the link to the electronic supplementary material.

Supplementary Material 1

## Data Availability

No datasets were generated or analysed during the current study.
