# Supplementary figures and images for: Restoration of posterior teeth by narrow diameter implants in hyperglycemic and normoglycemic patients – 4-year results of a case-control study
Source: Clin Oral Investig. 2024 Jun 22;28(7):392. doi: 10.1007/s00784-024-05786-0 (PMC11192651; doi:10.1007/s00784-024-05786-0)

## Slide 1
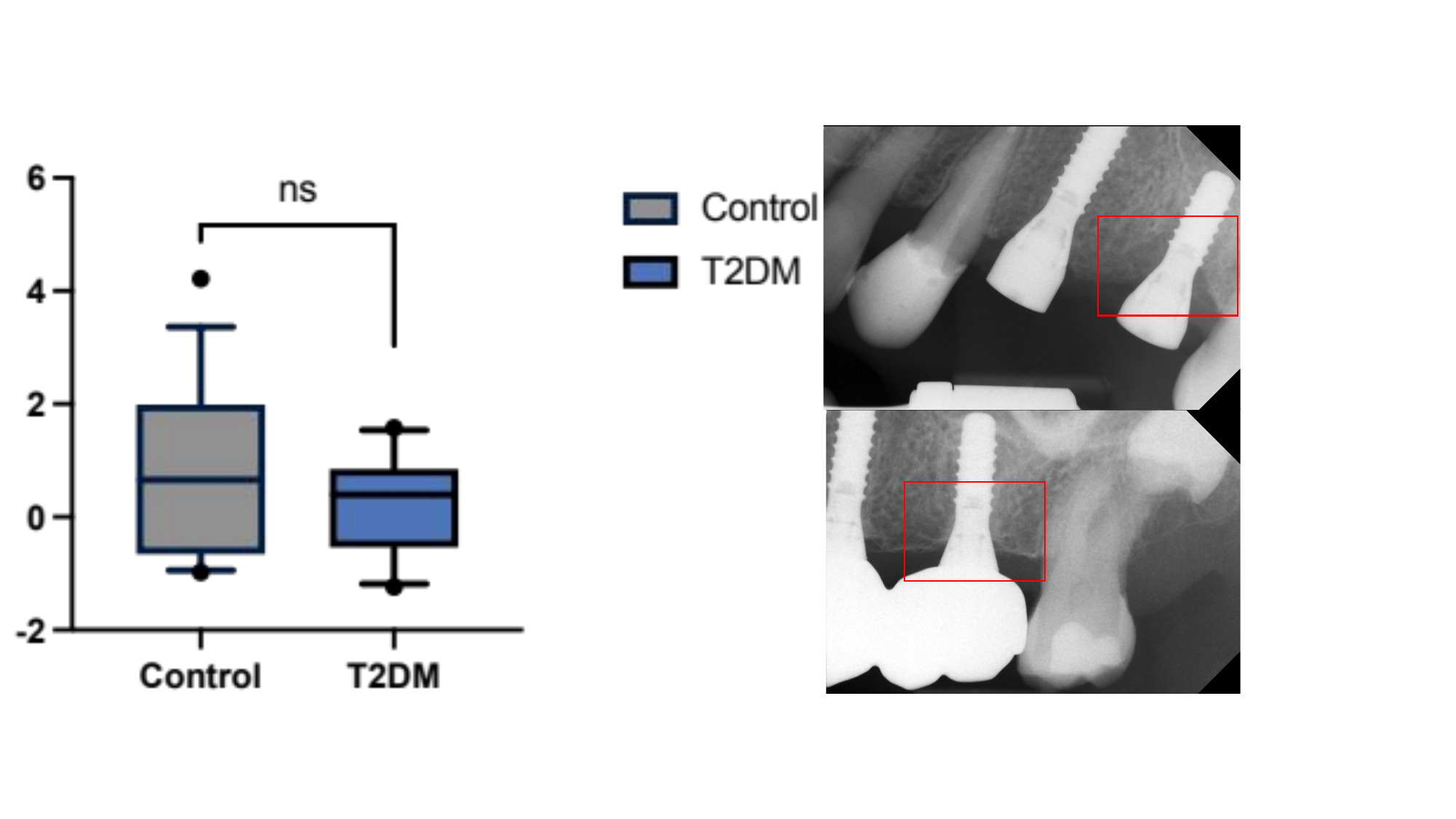

Supplement: Supplementary file 1 — Supplementary Material 1 [file 784_2024_5786_MOESM1_ESM.pptx]
